# Impact of the Histidine-Containing Phosphocarrier Protein HPr on Carbon Metabolism and Virulence in *Staphylococcus aureus*

**DOI:** 10.3390/microorganisms9030466

**Published:** 2021-02-24

**Authors:** Linda Pätzold, Anne-Christine Brausch, Evelyn-Laura Bielefeld, Lisa Zimmer, Greg A. Somerville, Markus Bischoff, Rosmarie Gaupp

**Affiliations:** 1 Institute of Medical Microbiology and Hygiene, Saarland University, D-66421 Homburg, Germany; Linda.Paetzold@uks.eu (L.P.); anne-christine.brausch@web.de (A.-C.B.); evelyn_kirch@yahoo.de (E.-L.B.); zimmer.lisa@yahoo.de (L.Z.); rosmariegaupp@gmail.com (R.G.); 2 School of Veterinary Medicine and Biomedical Sciences, University of Nebraska, Lincoln, NE 68588, USA; gsomerville3@unl.edu

**Keywords:** *Staphylococcus aureus*, physiology, metabolism, carbon catabolite repression, CcpA, HPr

## Abstract

Carbon catabolite repression (CCR) is a common mechanism pathogenic bacteria use to link central metabolism with virulence factor synthesis. In gram-positive bacteria, catabolite control protein A (CcpA) and the histidine-containing phosphocarrier protein HPr (encoded by *ptsH*) are the predominant mediators of CCR. In addition to modulating CcpA activity, HPr is essential for glucose import via the phosphotransferase system. While the regulatory functions of CcpA in *Staphylococcus aureus* are largely known, little is known about the function of HPr in CCR and infectivity. To address this knowledge gap, *ptsH* mutants were created in *S. aureus* that either lack the open reading frame or harbor a *ptsH* variant carrying a thymidine to guanosine mutation at position 136, and the effects of these mutations on growth and metabolism were assessed. Inactivation of *ptsH* altered bacterial physiology and decreased the ability of *S. aureus* to form a biofilm and cause infections in mice. These data demonstrate that HPr affects central metabolism and virulence in *S. aureus* independent of its influence on CcpA regulation.

## 1. Introduction

Carbon catabolite repression (CCR) is a common regulatory mechanism of bacteria to coordinate central metabolism with available carbon source(s) [1]. By modulating transcription of genes encoding proteins involved in the import and catabolism of carbon metabolites, bacterial CCR facilitates the efficient use of available carbon sources [1]. In pathogenic bacteria, regulators of CCR often affect transcription of virulence factors that are important for the exploitation of host-derived nutrient sources [2].

*Staphylococcus aureus* is a gram-positive opportunistic pathogen and a frequent cause of nosocomial infections in which central metabolism and infectivity are linked by numerous regulatory factors, including the catabolite control proteins A (CcpA) and E (CcpE), CodY, Rex, RpiRc, and SrrAB [3]. CcpA, a member of the GalR-LacI repressor family [4], is thought to be the major factor regulating CCR in *S. aureus* by binding catabolite-responsive element (*cre*) sequences of target genes [5]. Depending on the *cre* sequence location in the promotor region, the binding of CcpA results in either activation or repression of transcription [6]. Studies using *Bacillus megaterium* and *Streptococcus pyogenes* demonstrated that the binding affinity of CcpA for *cre* sites is low, but can be increased drastically by complex formation with the histidine-containing phosphocarrier protein (HPr), encoded by *ptsH* [7,8]. Electrophoretic mobility shift assays suggest this is also true in *S. aureus* [6], although CcpA can also bind to *cre* sites in the absence of HPr [9]. Activity of HPr is dependent on at least two phosphorylation sites, namely amino acids histidine 15 (His-15) and serine 46 (Ser-46) [1]. For complex formation with CcpA, HPr must be phosphorylated on Ser-46 [7]. This ATP-requiring process is catalyzed by the HPr-kinase/phosphorylase (HPrK/P), which is regulated in a dose-dependent manner by the glycolytic intermediate fructose-1,6-bisphosphate (FBP) [10]. For this reason, the amount of Ser-46 phosphorylated HPr (P-Ser-HPr) is closely connected with glycolytic activity of the cell and the uptake of sugars. Sugar uptake in bacteria is predominantly mediated by the phosphotransferase system (PTS), consisting of three main components: HPr, enzyme I (EI), and enzyme II (EII) [11]. In a first step, HPr is phosphorylated at His-15 (P-His-HPr) by E1, using the glycolytic intermediate phosphoenolpyruvate as the phosphate donor. The phosphate group is transferred to the substrate by EII, which translocates and phosphorylates the sugar into the cell at the same time. Activated glucose, namely glucose 6-phosphate, then enters glycolysis [12]; hence, HPr connects glycolytic activity with CCR via its dual role in sugar uptake through the PTS and as an activator of CcpA [13,14].

Numerous genes have been identified to be regulated on the transcriptional level by CcpA in *S. aureus* [15,16,17]. In addition to genes/operons involved in carbon catabolism, the synthesis of factors associated with biofilm formation and virulence of *S. aureus* are also influenced by CcpA [6,15,16,17,18,19,20,21]. Specifically, CcpA promotes transcription of the *ica*-operon and *cidA* [19], encoding proteins needed for polysaccharide intercellular adhesion (PIA) synthesis and extracellular DNA release, respectively [22,23]. These observations are consistent with the fact that deletion of *ccpA* abrogates biofilm formation under glucose-rich conditions. [19]. Furthermore, inactivation of *ccpA* in *S. aureus* reduces the formation of liver and skin abscesses in mouse models of infection [6,24,25]. Taken together, these observations demonstrate the linkage between CcpA, glucose catabolism, and virulence in *S. aureus*; however, the function of HPr remains largely unknown. Here, we characterize the function of HPr of *S. aureus* in the context of carbon metabolism, growth kinetics, biofilm formation, and in vivo infectivity in different murine infection models.

## 2. Materials and Methods

### 2.1. Bacterial Strains and Plasmids

The bacterial strains and plasmids used in this study are listed in Table 1. All mutant strains generated for this study were confirmed by sequencing of the affected region, and by assessing gene transcription by quantitative real-time reverse transcriptase PCR (qRT-PCR).

### 2.2. Bacterial Growth Conditions

*S. aureus* strains were grown in tryptic soy broth (TSB) containing 0.25% (*w*/*v*) glucose (BD, Heidelberg, Germany) or on TSB plates containing 1.5% agar (TSA). Antibiotics were only used for strain construction and phenotypic selection at the following concentrations: tetracycline, 2.5 µg/mL; erythromycin, 2.5 µg/mL; kanamycin, 15 µg/mL; and chloramphenicol, 10 µg/mL. Bacteria from overnight cultures were diluted in pre-warmed TSB to an optical density at 600 nm (OD_600_) of 0.05. All bacterial cultures were incubated at 37 °C and 225 rpm with a flask-to-medium ratio of 10:1. Samples for determination of the OD_600_, pH, and metabolites were taken every hour. The growth rate (*µ*) of *S. aureus* strains was calculated by the formula (ln *OD*_2_–ln *OD*_1_)/(*t*_2_–*t*_1_), with *OD*_1_ and *OD*_2_ being the OD calculated from the exponential growth phase at time *t*_1_ and *t*_2_, respectively. The generation time of each strain was determined using the formula ln 2/*µ*.

### 2.3. Mutant Construction

For the *S. aureus ptsH* deletion mutants, 1.4- and 1.1-kb fragments (nucleotides 1053472-1054838 and 1055049-1056169 of GenBank accession no. AP009351.1, respectively), containing the flanking regions of the *ptsH* open reading frame (ORF), were amplified by PCR from chromosomal DNA of *S. aureus* strain Newman using primer pairs MBH-94/MBH-112 and MBH-113/MBH-114, respectively (Supplementary Table S1). The PCR products were digested with KpnI/EcoRI and BamHI/XbaI, respectively, and cloned together with the EcoRI/BamHI-digested lox66-*aphAIII*-lox71 resistance cassette obtained from pBT *lox-aph* [26] into KpnI/XbaI-digested suicide vector pBT [27] to generate plasmid pBT *ptsH* KO. Plasmid pBT *ptsH* KO was propagated in *E. coli* strain DC10B [28] and subsequently electroporated directly into *S. aureus* strain Newman to obtain strain Newman Δ*ptsH*-*aph*, in which nucleotides 8 to 225 of the 267-bp spanning *ptsH* ORF were replaced by the lox66-*aphAIII*-lox71 cassette by allelic replacement. The deletion of *ptsH* in Newman Δ*ptsH*-*aph* was confirmed by PCR, and the strain was then used as a donor for transducing the lox66-*aphAIII*-lox71 tagged *ptsH* deletion into *S. aureus* strains SA113 and RN4220. Resistance marker-free Δ*ptsH*::lox72 derivatives were constructed by treatment with a Cre recombinase expressed from the temperature-sensitive vector pRAB1 [29], which was subsequently removed from the *aphIII*-cured derivatives by culturing the strains at 42 °C.

For the cis-complementation of the Δ*ptsH*::lox72 mutants, a 1-kb fragment (nucleotides 1055996-1056973 of GenBank accession no. AP009351.1) of the C-terminal region of the *ptsI* ORF and the annotated terminator region of the *ptsHI* operon was amplified by primers MBH-427/MBH-428 (Supplementary Table S1), digested with EcoRI/KpnI and cloned into EcoRI/KpnI-digested suicide vector pBT [27] to generate plasmid pBT ‘*ptsI*. The plasmid was electroporated into *S. aureus* strain RN4220, and a tetracycline-resistant RN4220 derivative that integrated pBT ‘*ptsI* in its chromosome at the *ptsI* locus was used as donor to phage-transduce the *tet*(L)-tagged *ptsI* allele into Nm Δ*ptsH* and SA113 Δ*ptsH*, respectively, thereby replacing the *ptsH::*lox72 deletion with the *ptsI*::pBT ‘*ptsI* genomic region containing a functional *ptsHI* operon.

For the construction of *S. aureus ptsH* variants harboring a T to G exchange of nucleotide 136 of the *ptsH* ORF (termed ptsH*), 0.6-kb and 1.1-kb fragments, containing either the promoter region of *ptsH* and the N-terminal part of the *ptsH* ORF (nucleotides 1054446-1054998 of GenBank accession no. AP009351.1) or the C-terminal part of the *ptsH* ORF and an N-terminal fragment of the *ptsI* ORF (nucleotides 1054976-1056121 of GenBank accession no. AP009351.1) were amplified by PCR from chromosomal DNA of *S. aureus* strain Newman using primer pairs MBH-484/MBH-485 and MBH-86/MBH-20, respectively (Supplementary Table S1). Primer MBH-484 contains a non-complementary base that introduces a point mutation in the PCR fragment leading to the T136G exchange of the *ptsH* ORF. Both PCR products were digested with StyI and subsequently ligated with T4 DNA-ligase. The ~1.7-kb ligation product was gel-purified, digested with KpnI/PstI, and cloned into KpnI/PstI-digested pBT to generate plasmid pBT *ptsH1* (Table 1). Presence of the T136G exchange in ptsH* harbored by plasmid pBT *ptsH1* was confirmed by sequencing, the plasmid propagated in *E. coli* strain DH5α, electroporated into RN4220 Δ*ptsH*, and selected for tetracycline-resistance. A tetracycline-resistant RN4220 derivative that integrated pBT *ptsH1* at the *ptsHI* locus was used as donor to transduce the *tet*(L)-tagged ptsH* allele into the Δ*ptsH* mutants.

*S. aureus* double mutants lacking *ptsH* and *ccpA* were created by transducing the *tet*(L)-tagged *ccpA* deletion of MST14 into Δ*ptsH* derivatives.

### 2.4. RNA Isolation and Purification, cDNA Synthesis and qRT-PCR

*S. aureus* strains were cultivated in TSB as described above. Bacterial pellets were collected after 2 h and 8 h of incubation by centrifugation at 5000 rpm at 4 °C for 5 min, and immediately suspended in 100 µL ice-cold TE-buffer (10 mM Tris-HCl, 1 mM EDTA, pH 8). Bacteria were disrupted, total RNA isolated, transcribed into cDNA, and qRT-PCRs carried out as described previously [33] using the primers listed in Supplementary Table S1. Transcriptional levels of target genes were normalized against the mRNA concentration of housekeeping gene *gyrB* according to the 2^−ΔCT^ method.

### 2.5. Measurement of pH, Glucose, Acetate, and Ammonium in Culture Supernatants

Aliquots (1.5 mL) of bacterial cultures were centrifuged for 2 min at 10,000× *g*, and supernatants were removed, pH measured, and stored at −20 °C until further use. Glucose, acetate, and ammonia concentrations were determined with kits purchased from R-Biopharm (Pfungstadt, Germany) and used according to the manufacturer’s directions. The metabolite concentrations were measured from at least three independent experiments.

### 2.6. Biofilm Assays

Biofilm formation under static conditions was assessed as described [19]. Briefly, overnight cultures were diluted to an OD_600_ of 0.05 in fresh TSB medium supplemented with glucose to a final concentration of 0.75 % (*w*/*v*), and 200 µL of the cell suspension was used per well to inoculate sterile, flat-bottom 96-well polystyrene microtiter plates (BD). After incubation for 24 h at 37 °C without shaking, the plate wells were washed twice with phosphate-buffered saline (pH 7.2) and dried in an inverted position. Adherent cells were safranin-stained (30 sec with 0.1% safranin; Merck, Darmstadt, Germany) and the absorbance of stained biofilms was measured at 490 nm after resolving the stain with 100 µL 30 % (*v*/*v*) acetic acid, using a microtiter plate reader (Victor^2^ 1420 Multilabel Counter; Perkin Elmer, Rodgau, Germany).

Biofilm formation under flow conditions was performed as described [34], with minor modifications: Bacteria from overnight cultures were diluted to an OD_600_ of 0.05 in fresh TSB medium supplemented with glucose to a total concentration of 0.75% (*w*/*v*) and cultivated for 2 h at 37 °C with shaking at 150 rpm. Flow cells (Stovall Life Science) were filled with pre-warmed TSB medium supplemented with glucose to a total concentration of 0.75% (*w*/*v*), attached to a peristaltic pump (Ismatec REGLO Digital; Postnova, Landsberg am Lech, Germany) and inoculated with 0.5 mL of the bacterial cultures. Thirty minutes after inoculation, the flowrate was set to 0.5 mL/min and chamber. Biofilm formation was visually documented at different times.

For the assessment of biofilm formation on medical devices under dynamic conditions, peripheral venous catheter (PVC, Venflon Pro Safety 18 G; BD) fragments of 1 cm length were placed into reaction tubes filled with 1 mL of TSB and inoculated with 5 × 10^5^ CFU of TSB-washed bacterial cells obtained from exponential growth phase (inoculation of TSB from overnight cultures to an OD_600_ of 0.05 and incubation for 2.5 h at 37 °C and 225 rpm). The PVC fragments were incubated under non-nutrient limited conditions for five days at 37 °C and 150 rpm, and the media were replaced with fresh media every 24 h. PVC fragments were placed five days post inoculation into fresh reaction tubes filled with 1 mL of TSB, biofilms were detached from the catheter surface and resolved by sonification (50 watt for 5 min) followed by 1 min of vortexing. CFU rates and biomasses of resolved biofilms and culture supernatants at day five post inoculation were determined by plate counting and OD_600_ measurements, respectively.

### 2.7. Primary Attachment Assay on Polystyrene

The primary attachment of bacterial cells to polystyrene surfaces was performed as described [35], with minor modifications. Briefly, bacteria from the exponential growth phase (inoculation of TSB from overnight cultures to an OD_600_ of 0.05 and incubation for 2.5 h at 37 °C and 225 rpm) were diluted in TSB to 3000 CFU/mL. 100 µL of the bacterial inoculum was poured onto polystyrene petri dishes (Sarstedt, Nümbrecht, Germany) and incubated under static conditions at 37 °C for 30 min. After incubation, petri dishes were rinsed gently three times with 5 mL of sterile PBS (pH 7.5), and subsequently covered with 15 mL of TSB containing 0.8% agar maintained at 48 °C. Plates were incubated at 37 °C for 24 h. Bacterial attachment to polystyrene was defined as the number of CFU remaining on the petri dish bottom after washing compared to the number of CFU remaining on the petri dish bottom without washing.

### 2.8. Animal Models

All animal experiments were performed with approval of the local State Review Board of Saarland, Germany (project identification codes 60/2015 [approved 21.12.2015], and 34/2017 [approved 09.11.2017]), and conducted following the national and European guidelines for the ethical and human treatment of animals. PBS-washed bacterial cells obtained from exponential growth phase cultures were used as inoculum.

For the murine abscess model, infection of animals was carried out as described [33], with minor modifications; specifically, 8- to 12- week-old female C57BL/6N mice (Charles River, Sulzfeld, Germany) were anesthetized by isoflurane inhalation (3.5%; Baxter, Unterschleißheim, Germany) and 100 µL bacterial suspension containing 5 × 10^7^ CFU were administered intravenously by retro bulbar injection. Immediately after infection, animals were treated with a single dose of carprofen (5 mg/kg; Zoetis, Berlin, Germany). Behavior and weight of mice was monitored daily, and four days post-infection, mice were sacrificed, and livers and kidneys were removed. The bacterial loads in liver and kidney tissues were determined by homogenization of weight-adjusted organs in PBS (pH 7.4), followed by serial dilutions on sheep blood agar plates and plate counting after 24 h incubation at 37 °C.

For the *S. aureus* based murine foreign body infection model, implantation of catheter fragments and infection of animals was carried out as described [36], with minor modifications: 8- to 12- week-old female C57BL/6N mice (Charles River) were anesthetized by intraperitoneal injection of 0.05 mg/kg body weight fentanyl (Hexal, Holzkirchen, Germany), 5 mg/kg midazolam (Hameln Pharma Plus, Hameln, Germany) and 0.5 mg/kg medetomidine (Orion Pharma, Hamburg, Germany). After treatment with a dose of carprofen (5 mg/kg, Zoetis), the animals were shaved with an animal trimmer (BBraun, Melsungen, Germany) and depilated with asid-med hair removal cream (Asid Bonz, Herrenberg, Germany) on both flanks. The depilated skin was disinfected with ethanol (70%) and 1 cm catheter fragments (PVC, 14G, Sarstedt) were implanted subcutaneously and inoculated with 1 × 10^4^ CFU of the respective *S. aureus* strains. Wounds were closed with staples (Fine Science Tools, Heidelberg, Germany) and anesthesia was antagonized with 1.2 mg/kg body weight naloxone (Inresa, Freiburg im Breisgau, Germany), 0.5 mg/kg flumazenil (Inresa) and 2.5 mg/kg atipamezole (Orion Pharma). Behavior and weight of the animals was monitored daily. Ten days post infection, animals were sacrificed, edema sizes were measured and photo documented, and catheter fragments with surrounding tissue were harvested for microbial analyses. Excised tissues were homogenized in 1 mL TSB with a hand disperser (POLYTRON PT 1200 E; Kinematica, Eschbach, Germany), and biofilms were detached from the PVC fragments and resolved by sonification (50 watt for 5 min) followed by vortexing (1 min). CFU rates in tissue and of biofilm formed on the catheter were determined by plating serial dilutions on sheep blood agar plates and plate counting after 24 h of incubation at 37 °C.

### 2.9. Statistical Analyses

The statistical significance of changes between groups was assessed by one-way ANOVA followed by Holm-Sidak’s post-hoc tests for experiments containing ≥ 5 biological replicates using the GraphPad software package Prism 6.01 (San Diego, CA 92108, USA). *p* values < 0.05 were considered statistically significant.

## 3. Results and Discussion

### 3.1. Growth, pH Characteristics, and Metabolite Profiles Differ between ptsH and ccpA Mutants

**Figure 1:**
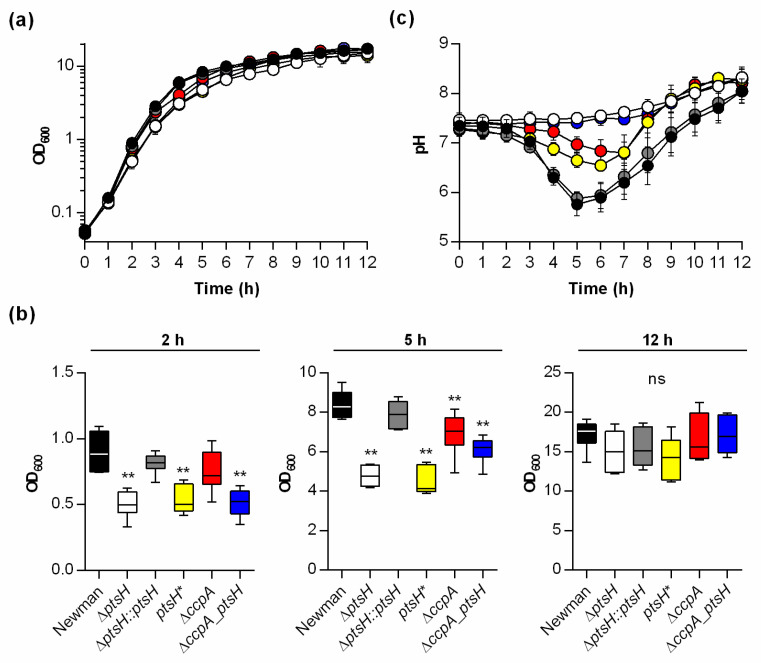
Impact of *ptsH* and/or *ccpA* on growth and pH profiles of *S. aureus* TSB cultures. Bacteria were inoculated to an OD_600_ of 0.05 in TSB and cultured aerobically at 37 °C and 225 rpm. OD_600_ (**a**,**b**) and pH measurements (**c**) of the culture media were determined hourly. Symbols represent: strains Newman (black symbols), Nm *ptsH* (white symbols), Nm *ptsH::ptsH* (grey symbols), Nm ptsH* (yellow symbols), Nm *ccpA* (red symbols), and Nm *ccpA_ptsH* (blue symbols). The results are the mean ± SD of at least five independent experiments. (**b**) OD_600_ readings of the cell cultures at 2, 5, and 12 h of growth, respectively. The data are presented as box and whisker plot showing the interquartile range (25–75%, box), the median (horizontal line), and the standard deviation (bars) of 5–6 independent experiments. **, *p* < 0.01 (one-way ANOVA and Holm-Sidak’s multiple comparison test. Only differences between Newman and mutants are shown).

To determine if inactivation of *ptsH* in *S. aureus* leads to changes in growth and carbon catabolism, mutants were constructed in the *S. aureus* laboratory strain Newman (Table 1) and growth and physiology were assessed (Figure 1). In detail, a mutant lacking *ptsH* (Δ*ptsH*) was constructed and cis-complemented (*ptsH::ptsH*), and a Δ*ccpA ptsH* double mutant was created (*ccpA_ptsH*). In addition, a *ptsH* mutant harboring a point mutation in the *ptsH* gene (T136G) leading to the substitution of serine to alanine at position 46 of HPr (HPr-S46A) was constructed (ptsH*). The phosphorylation at this amino acid represents a known prerequisite for HPr to activate CcpA in other gram-positive bacteria (14), while its activity in the phosphotransferase uptake system (PTS) should be unaffected. The parental strain Newman and the cis-complemented *ptsH* derivative displayed similar growth characteristics and comparable generation times, respectively. In contrast, all *ptsH* mutants (*ptsH*, *ccpA_ptsH*, and ptsH*) had reduced growth rates in the exponential (1–3 h) and the transition phase (4–6 h) relative to the wild type (Figure 1 and Table 2). Interestingly, the growth rate of the isogenic *ccpA* deletion mutant was only slightly diminished relative to that of strain Newman (Figure 1 and Table 2), and differed significantly from the wild type only during the transition phase. After 12 h of cultivation, growth yields were comparable for all strains (Figure 1b), suggesting that neither the lack of CcpA nor HPr has a clear long-term effect on biomass production of *S. aureus* cultured in rich medium. This is in line with earlier findings regarding CcpA [15].

To assess the bacterial acid production during growth, the pH of culture supernatants was measured over time (Figure 1c). The pH of culture supernatants from wild type and cis-complemented *ptsH* mutant cultures were similar. In contrast, pH values of culture supernatants from the *ptsH* and *ccpA_ptsH* deletion mutants indicated that little acid was produced during growth. The pH profiles of the ptsH* and *ccpA* mutant cultures were between these two extremes but indicated that acidic end-products were produced and consumed during growth. Taken together, these data indicate that inactivation of *ptsH*, or interference with HPr phosphorylation, delays growth and medium acidification to a greater extent than does deletion of *ccpA*. In addition, the small differences in physiological parameters (i.e., growth and pH kinetics) between the *ptsH* and the *ccpA_ptsH* mutants and between the *ccpA* and ptsH* mutants indicate additional, CcpA-independent functions of HPr.

**Figure 2:**
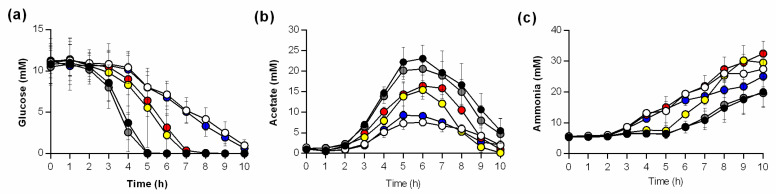
Impact of *ptsH* and/or *ccpA* on glucose consumption, acetate and ammonia production of *S. aureus* Newman during in vitro growth. *S. aureus* strains Newman (black symbols), Nm *ptsH* (white symbols), Nm *ptsH::ptsH* (grey symbols), Nm ptsH* (yellow symbols), Nm *ccpA* (red symbols), and Nm *ccpA_ptsH* (blue symbols) were cultivated in TSB, and glucose (**a**), acetate (**b**), and ammonia (**c**) concentrations in culture supernatants were determined hourly. Results are presented as the average and standard deviation of at least three independent experiments.

To get an idea about the metabolic processes that are active in Newman wild type and mutant cells cultured in TSB, the concentrations of glucose, acetate, and ammonia were determined in culture supernatants over time (Figure 2). Strain Newman and the cis-complemented *ptsH* mutant (*ptsH::ptsH*) depleted all available glucose in the medium within the first 5 h of cultivation (Figure 2a). In contrast, glucose depletion in Δ*ptsH* and Δ*ccpA_ptsH* mutant cultures was severely delayed, and low concentrations of glucose were still detectable in culture supernatants even after 10 h of growth. In Δ*ccpA* and ptsH* mutant cultures, glucose levels decreased slower than in wild type cultures, and no glucose was detectable after 7 h of growth (Figure 2a).

When *S. aureus* is cultured aerobically in a glucose-containing medium, cells produce and secrete acetate as long as glucose is available [37]. Consistent with this fact, increasing acetate concentrations in the culture supernatants were observed during the first 5–6 h of growth for all strains (Figure 2b). However, while all strains accumulated acetate in the medium, the maximum concentrations differed; specifically, the wild type and the cis-complemented *ptsH* derivative accumulated up to 22 mM of acetate. In contrast, supernatants from Δ*ptsH* and Δ*ccpA_ptsH* mutant strain cultures had approximately one-third of the concentration of that from the wild type strain. Similar to that seen in the pH profiles (Figure 1c), the acetate profiles of ptsH* and Δ*ccpA* mutant cultures centered in between those two extremes (Figure 2b). At 7–8 h post inoculation, acetate levels decreased in the supernatants of all cultures, irrespective of the fact that glucose was present in Δ*ptsH* and Δ*ccpA_ptsH* mutant cultures (Figure 2a).

*S. aureus* also utilizes amino acids as carbon sources for growth, a process that requires deamination of the amino acids, resulting in the secretion of ammonia into the culture supernatant [20]. The uptake and catabolism of amino acids in *S. aureus* is subject to CCR [20]. While glucose was present in the medium, ammonia levels remained low in the wild type and *ptsH::ptsH* culture supernatants, followed by a steady increase in the ammonia concentrations (Figure 2c). In contrast, cultures of strains Nm *ptsH* and Nm *ccpA_ptsH* began to accumulate ammonia beginning at 3 h of cultivation. Interestingly, the ammonia concentration in the supernatant of the Δ*ccpA* mutant closely resembled that of the *ptsH* deletion mutants, while the ptsH* mutant resembled the late induction of the wild type strain (Figure 2c).

Taken together, these data show that the inactivation of *ptsH* or *ccpA* results in distinct differences in glucose consumption, acetate accumulation and reutilization, and ammonia secretion in *S. aureus*. Furthermore, the exchange of an amino acid critical for the interaction of HPr with CcpA in the ptsH* mutant resulted in metabolite profiles (i.e., glucose and acetate) comparable to the Δ*ccpA* mutant, while some alterations in the growth profile, generation time, and ammonia secretion were observed. Importantly, after 12 h of growth, the biomass of *S. aureus* Newman was independent of *ccpA* and *ptsH*, suggesting that *S. aureus* has other means to utilize carbon sources in the growth medium. Specifically, *ptsH* mutants were able to utilize glucose from the growth medium—although much slower than the wild type—demonstrating that *S. aureus* can transport glucose independent of the group translocation PTS [38]. A likely compensatory transporter would be one of the many ATP binding cassette transporters identified in *S. aureus* [39].

### 3.2. Inactivation of ptsH and/or ccpA Alters Transcription of TCA Cycle and Virulence Factor Genes

**Figure 3:**
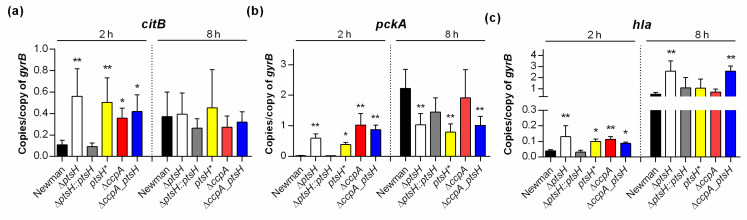
Effect of *ptsH* and/or *ccpA* mutations on the transcription of *S. aureus*. Newman wild type and mutant cells were cultured aerobically in TSB, as outlined in Materials and Methods. Cells were harvested at the time points indicated, total RNAs isolated, and qRT-PCRs performed for *citB* (**a**), *pckA* (**b**), and *hla* (**c**). Transcripts were quantified in reference to the transcription of gyrase B. Data are presented as mean + SD of five biological replicates. *, *p* < 0.05; **, *p* < 0.01 (one-way ANOVA and Holm-Sidak’s multiple comparison test; only differences between Newman and mutants are shown).

CcpA is known to affect transcription of a large number of central carbon metabolism and virulence genes [15,16,17]. For this reason, the effect of *ptsH* deletion on transcription of genes regulated by CcpA such as *citB* (encoding the TCA cycle key enzyme aconitase), *pckA* (encoding the gluconeogenesis key enzyme phosphoenolpyruvate carboxykinase), and *hla* (encoding α-hemolysin) was assessed. Specifically, mRNA levels were determined in cells from the exponential (i.e., 2 h) and post-exponential growth phases (i.e., 8 h) by qRT-PCR (Figure 3).

Consistent with our previous observation that *S. aureus* transcription of *citB* and *pckA* is repressed by CcpA when cultivated with glucose [16], deletion of *ccpA* significantly increased the level of *citB* and *pckA* mRNA in exponential growth phase cells relative to wild type cells (Figure 3a,b). As expected, in the post-exponential growth phase, comparable *citB* and *pckA* transcript levels were observed in the Δ*ccpA* mutant and the wild type. Similar to the Δ*ccpA* mutant, exponential growth phase cells of the *ptsH* mutants (Δ*ptsH*, ptsH*, and Δ*ccpA_ptsH*) had comparable *citB* and *pckA* mRNA levels, while the cis-complemented *ptsH* derivative (*ptsH::ptsH*) had *citB* and *pckA* transcript levels comparable to the wild type strain. In contrast to exponential growth phase cultures, all three *ptsH* mutants produced significantly lower levels of *pckA* mRNA than the wild type at 8 h, suggesting that *pckA* transcription is affected by HPr at later growth stages in a way that is independent of CcpA. This differed from the results for *citB* in which all *ptsH* mutants (Δ*ptsH*, ptsH*, and Δ*ccpA_ptsH*) had comparable transcript levels to that of the Δ*ccpA* mutant and the wild type after 8 h of growth. The fact that the ptsH* mutant produced *pckA* transcript levels similar to the *ptsH* mutant, but not similar to the *ccpA* mutant suggests that HPr phosphorylated at serine 46 acts in part independent of CcpA. This observation is consistent with that found in other gram-positive bacteria, where the serine 46-phosphorylated HPr exerted effects on CCR via CcpA and inducer exclusion [40]. However, it cannot be excluded that differences in *pckA* transcription between the ptsH* and Δ*ccpA* mutants are due to differences in protein stability. The reason why protein stability cannot be excluded is because phosphorylation of the *B. subtilis* HPr homolog at Ser-46 stabilized the protein [41], while a serine to alanine exchange of Ser-46 in the *E. coli* HPr homolog was found to decrease the stability of the protein [42].

CcpA represses transcription of *hla* during the exponential growth phase when bacteria are cultured in presence of glucose [15,16]. Similarly, levels of *hla* mRNA from the exponentially growing Δ*ccpA* mutant and all *ptsH* mutants (Figure 3c) were de-repressed, while the cis-complemented *ptsH* deletion mutant produced *hla* transcript levels that were comparable to the wild type. During the post-exponential growth phase, only the *hla* transcript levels of the Δ*ptsH* and the Δ*ccpA_ptsH* double mutant were significantly increased (Figure 3c), suggesting that HPr affects expression of α-hemolysin in a CcpA-dependent and -independent manner. Taken together, these data suggest that exponential growth phase *S. aureus* was cultured in the presence of glucose, HPr affects the transcription primarily via activation of CcpA, while in the post-exponential growth phase cells of *S. aureus*, HPr is likely to affect gene transcription by CcpA-independent mechanism(s).

### 3.3. Impact of ptsH Deletion on Biofilm Formation of S. aureus SA113

**Figure 4:**
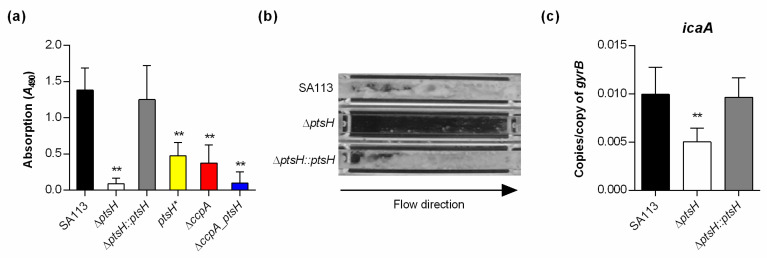
Mutations of *ptsH* affect biofilm formation of *S. aureus* under static and flow conditions. (**a**) Biofilm growth of *S. aureus* strains in a static 96-well microplate assay. The data show the mean + SD of five biological replicates. (**b**) Flow cell chambers were inoculated with *S. aureus* strains as indicated, allowed to attach to the surfaces for 30 min, and incubated under constant flow for 24 h. The results shown are representative of two independent experiments. (**c**) Effect of the *ptsH* mutation on the transcription of *icaA* in *S. aureus* strain SA113. Cells of SA113, the Δ*ptsH* mutant, and the cis-complemented *ptsH::ptsH* derivative were cultured aerobically in TSB. After 2 h of growth, cells were harvested, total RNAs isolated, and qRT-PCRs performed for *icaA*. Transcripts were quantified in reference to gyrase B mRNA. Data are presented as mean + SD of five biological replicates. **, *p* < 0.01 (one-way ANOVA and Holm-Sidak’s multiple comparison test; only differences between SA113 and mutants are shown).

CcpA is important for polysaccharide intercellular adhesin (PIA)-dependent biofilm formation by staphylococci under glucose-rich in vitro conditions [19,24,43]. The importance of HPr on sugar import and gene regulation suggests that HPr might influence biofilm formation of *S. aureus*. Strain Newman is a weak biofilm producer in glucose-rich medium under in vitro conditions [34], hence we transduced the *ptsH* mutations into *S. aureus* strain SA113, which forms a strong biofilm under these conditions [19,22]. The ability of SA113 mutant strains were analyzed using a semi-quantitative static biofilm assay (Figure 4a) and in biofilm flow cells (Figure 4b). 

Under static conditions, the Δ*ptsH* and Δ*ccpA_ptsH* mutants of SA113 displayed drastic decreases in their biofilm formation capacities on polystyrene surfaces, whereas the cis-complemented derivative (*ptsH::ptsH*) formed biofilms that were comparable to the ones seen with the wild type (Figure 4a). Deletion of *ccpA* or the S46A mutation of HPr in SA113 (ptsH*) also significantly reduced biofilm formation, however, not to the extent seen with the Δ*ptsH* mutant, supporting our hypothesis that a functional PTS is important for *S. aureus* to form a biofilm in this type of assay. In the flow chamber assay, the Δ*ptsH* mutant failed to produce a clear biofilm within the microchannel after 24 h of constant flow, while both, the wild type and the cis-complemented *ptsH* derivative, almost completely filled the microchannel with biomass (Figure 4b), suggesting that HPr is also important for biofilm formation under shear flow. To exclude that the latter phenotype was caused by a decreased capacity of the Δ*ptsH* mutant to attach to the microchannel surface, the primary attachment capacities of the strains were determined. Here, no clear differences in attachment towards polystyrene surfaces were obtained for the strain triplet, suggesting that the observed lack of biofilm formation of the SA113 Δ*ptsH* mutant is likely due to a deficiency in biofilm maturation. To determine whether this effect might be due to a decreased capacity of the mutant to produce PIA, we assayed the transcription of *icaA*, which is part of the *icaADBC* polycistronic mRNA that encodes proteins needed for PIA synthesis [22]. Consistent with the reduced ability of the SA113 *ptsH* mutant to form a biofilm under static and flow conditions, we observed significantly decreased levels of *icaA* transcripts in the *ptsH* deletion mutant relative to the wild type and the cis-complemented mutant (Figure 4c). Together, these data suggest that HPr, in part, promotes biofilm formation of *S. aureus* by enhancing the expression of the PIA synthesis machinery.

**Figure 5:**
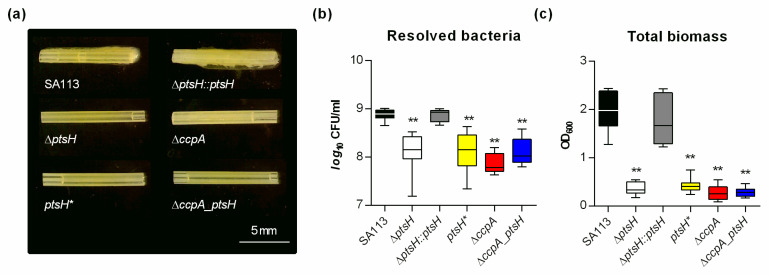
Inactivation of *ptsH* and/or *ccpA* reduces the biofilm formation capacity of *S. aureus* on medical devices. (**a**) Images of *S. aureus*-loaded catheter fragments at day 5 post inoculation (6.3-fold magnification). The results are representative of three independent experiments. (**b**,**c**) Colony forming units (CFU) and total biomass of detached biofilms were determined by plate counting (**b**) and measuring the OD_600_ of the TSB solutions (**c**). The data are presented as box and whisker plot showing the interquartile range (25–75%, box), the median (horizontal line), and the standard deviation (bars) of nine independent experiments. **, *p* < 0.01 (one-way ANOVA and Holm-Sidak’s multiple comparison test; only differences between SA113 and mutants are shown).

In a third biofilm assay intended to resemble the in vivo situation more closely, we studied the ability of SA113 and its derivatives to form biofilms on peripheral venous catheter (PVC) fragments under non-nutrient limited conditions (Figure 5). 

Using this assay, a strong biofilm was macroscopically detectable on catheter fragments inoculated with the wild type or the cis-complemented *ptsH* derivative at 5 days post inoculation (Figure 5a). In contrast, on catheter fragments inoculated with either the Δ*ptsH* mutant, the Δ*ccpA* mutant, the ptsH* mutant, or the Δ*ccpA_ptsH* double mutant, almost no biofilm was visible. These observations were further supported by CFU and OD_600_ determinations of TSB solutions harboring the detached biofilms (Figure 5b,c). A significant reduction in viable bacteria (~1 log) was observed on all fragments inoculated with mutants when compared to the wild type inoculated fragments (Figure 5b). Similar to the CFU data, the OD_600_ values were approximately 10-fold lower in the detached biofilms formed by the mutants (Figure 5c), suggesting that *ptsH* and *ccpA* deletions elicit rather comparable effects on the biofilm forming capacity of *S. aureus* on PVC surfaces under non-nutrient limited conditions.

### 3.4. HPr Contributes to Infectivity and Biofilm Formation of S. aureus SA113 in a Murine Foreign Body Infection Model

**Figure 6:**
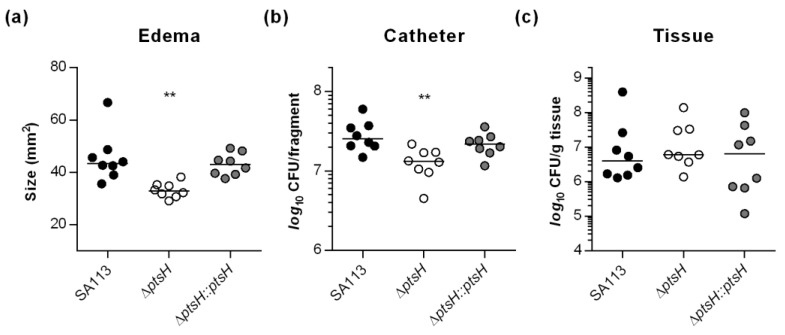
Inactivation of *ptsH* reduces the infectivity of *S. aureus* SA113 in a murine foreign body infection model. Catheter fragments were implanted subcutaneously into the back of normoglycemic mice and inoculated with cells of *S. aureus* strains SA113 (black symbols), its *ptsH* deletion mutant (white symbols), and the cis-complemented *ptsH* mutant (grey symbols), respectively (*n* = eight animals per group). Ten days post infection, animals were euthanized, edema sizes around the implanted catheters were measured (**a**), and the catheters and surrounding tissues were explanted. Bacterial loads from catheter detached biofilms (**b**) and in surrounding tissue homogenates (**c**) were determined by CFU counting. The data represent the values of every individual animal (symbols) and the median (horizontal line). **, *p* < 0.01 (one-way ANOVA and Holm-Sidak’s multiple comparison test).

CcpA is not required for biofilm formation of *S. aureus* and *S. epidermidis* on implanted catheter fragments in normoglycemic mice [24,43], but this did not address the function of HPr in vivo. In order to address this question, the ability of strains SA113, the Δ*ptsH* mutant, and the cis-complemented *ptsH* derivative to form biofilms on implanted catheter fragments was assessed in the murine foreign body infection model [36] with normoglycemic mice (Figure 6).

Mice challenged with the Δ*ptsH* mutant displayed a clear reduction in edema sizes around the implanted catheter fragments (Figure 6a) and a small but significant reduction (~2-fold) in detached bacteria (Figure 6b), when compared to animals infected with wild type bacteria or the cis-complemented *ptsH* derivative. In contrast, no significant differences in bacterial loads of tissues surrounding the catheter fragments were obtained (Figure 6c). These findings suggest that HPr, unlike CcpA, has a small but important function on the biofilm formation capacity of PIA producing *S. aureus* in normoglycemic mice. This difference is probably due to a reduced sugar uptake capacity of the *ptsH* mutant, which might interfere with the enhanced carbon and energy demand of *S. aureus* during biofilm maturation.

### 3.5. HPr and CcpA Are Both Required for Full Infectivity of S. aureus in a Murine Liver Abscess Model

**Figure 7:**
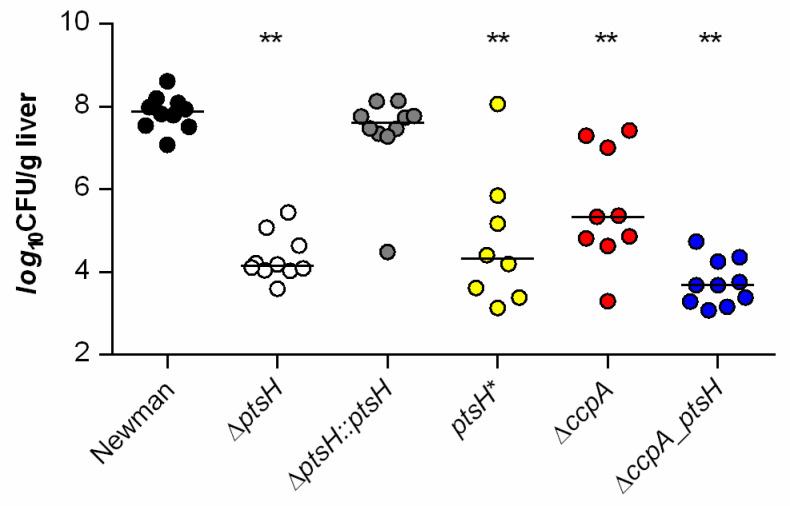
Inactivation of *ptsH* and/or *ccpA* results in decreased bacterial burden of *S. aureus* Newman in a murine abscess model. C57BL/6N mice were infected retro bulbar with 5 × 10^7^ CFU of *S. aureus* strains Newman (black symbols), its Δ*ptsH* (white symbols) and Δ*ccpA* (red symbols) mutants, the ptsH* mutant (yellow symbols), the Δc*cpA_ptsH* double mutant (blue symbols), and the cis-complemented *ptsH* derivative (grey symbols), respectively. Bacterial loads in liver tissue homogenates were determined four days post infection. The data display the median (horizontal lines) and individual values of every animal (dots; *n* = 8–10 animals per group). **, *p* < 0.01 (one-way ANOVA and Holm-Sidak’s multiple comparison test; only differences between Newman and mutants are shown).

The formation of liver abscesses is one of the clinical manifestations caused by *S. aureus* in which CcpA exerts a strong effect on disease progression in normoglycemic mice [6]. To determine how P-Ser-HPr affects infectivity of *S. aureus* in a murine liver abscess model, the bacterial loads in livers four days post infection were assessed (Figure 7).

Consistent with previous observations [6,24], we observed a nearly 3 log reduction in bacterial loads in liver tissue of C57BL/6 mice challenged with the *ccpA* mutant bacteria (median 2.2 × 10^5^ CFU/g tissue) relative to mice infected with the wild type strain (median 7.6 × 10^7^ CFU/g tissue). Importantly, a greater reduction in CFU/g liver was observed (~4 log; median 1.4 × 10^4^ CFU/g tissue), when mice were challenged with the Δ*ptsH* mutant. Infection of mice with the cis-complemented *ptsH* derivative resulted in a bacterial burden in the liver (median 4.2 × 10^7^ CFU/g tissue) comparable to that seen in wild type infected mice, demonstrating that the decreased CFU rates determined in liver tissues of Δ*ptsH* infected mice were due to the lack of HPr. Notably, mice challenged with the ptsH* mutant carrying the S46A exchange in HPr also caused an almost ~4 log reduction in bacterial loads in liver tissue (median 2.1 × 10^4^ CFU/g tissue), suggesting that both, the deletion of *ptsH* and a mutation of serine 46 of HPr, alter the virulence of *S. aureus* in this murine infection model in a CcpA-independent manner. The lowest CFU rates in liver tissues were observed when mice were challenged with the Δ*ccpA_ptsH* double mutant (median 4.8 × 10^3^ CFU/g tissue), suggesting that CcpA might also exert some effects on virulence of *S. aureus* in this infection model independently of HPr.

## 4. Conclusions

Central carbon metabolism and virulence factor synthesis are tightly linked in *S. aureus* and controlled by several transcription factors [3]. Notably, CcpA is the only transcription factor known to enhance infectivity of *S. aureus* [6,24,25], while other regulators such as CcpE, CodY, and RpiRc are thought to attenuate rather than to promote infectivity of this bacterium in mice [33,44,45,46,47]. We show here that HPr contributes positively to infectivity of *S. aureus* in mice, presumably by affecting central carbon metabolism and virulence factor synthesis in a CcpA-dependent and -independent manner. These effects are likely mediated through changes in sugar transport and carbon metabolization that alter biofilm formation [24]. It is also possible that HPr in *S. aureus* acts like the HPr homolog of *E. coli* to modulate quorum sensing by interacting with autoinducer-2 (AI-2) modifying factors [48]. Given the importance of HPr on biofilm formation and virulence in *S. aureus*, this phosphocarrier protein could be a promising drug target for the development of novel anti-staphylococcal compounds.

## Figures and Tables

**Table 1:** Strains and plasmids used in this study.

Strain	Description ^1^	Reference or Source
***S. aureus***		
Newman	Mouse pathogenic laboratory strain (ATCC 25904)	[30]
RN4220	NCTC8325-4 derivative, acceptor of foreign DNA	[31]
SA113	PIA-dependent biofilm producer (ATCC 35556), *agr rsbU*	[32]
Nm *ccpA*	MST14; Newman Δ*ccpA*::*tet*(L); Tc^R^	[15]
Nm *ptsH*	Newman Δ*ptsH*::lox72	This study
Nm *ptsH-aph*	Newman Δ*ptsH*::lox66-*aphaIII*-lox71; Kan^R^	This study
Nm *ptsH::ptsH*	Newman Δ*ptsH*::pBT *ptsH*; Tc^R^	This study
Nm ptsH*	Newman Δ*ptsH*::lox72 pBTptsH*; Tc^R^	This study
Nm *ccpA_ptsH*	Newman Δ*ccpA*::*tet*(L) Δ*ptsH*::lox72; Tc^R^	This study
RN4220 *ptsH*	RN4220 Δ*ptsH*::lox72	This study
SA113 *ccpA*	KS66; SA113 Δ*ccpA*::*tet*(L); Tc^R^	[19]
SA113 *ptsH*	SA113 Δ*ptsH*::lox72	This study
SA113 *ptsH::ptsH*	SA113 Δ*ptsH*::pBT *ptsH*; Tc^R^	This study
SA113 ptsH*	SA113 Δ*ptsH*::lox72 pBTptsH*; Tc^R^	This study
SA113 *ccpA*_*ptsH*	SA113 Δ*ccpA*::*tet*(L) Δ*ptsH*::lox72; Tc^R^	This study
***E. coli***		
DH5α	Cloning strain	Invitrogen
DC10B	Δ*dcm* in the DH10B background; Dam methylation only	[28]
**Plasmids**		
pBT	*S. aureus* suicide plasmid; *tet*(L)	[27]
pBT *lox-aph*	pBT derivative harboring lox66-*aphAIII*-lox71; *tet*(L), *aphIII*	[26]
pRAB1	Temperature sensitive *E. coli-S. aureus* shuttle plasmid, expression of *cre* in staphylococci; *cat*, *bla*	[29]
pBT ‘*ptsI*	pBT derivative harboring a C-terminal *ptsI* fragment; *tet*(L)	This study
pBT *ptsH1*	pBT derivative harboring a T136G *ptsH* variant; *tet*(L)	This study
pBT *ptsH* KO	pBT derivative harboring the genomic regions flanking *ptsH* and lox66-*aphAIII*-lox71 of pBT *lox-aph*; *aphIII,* *tet*(L)	This study

^1^ Kan^R^, kanamicin-resistant; PIA, polyintercellular adhesin; Tc^R^, tetracycline-resistant.

**Table 2:** Generation time of *S. aureus* strains cultivated in TSB under aerobic conditions.

Strain	Generation Time (min) ^1^	*p* Value ^2^
Newman	28.6 ± 1.9	
Nm *ptsH*	34.8 ± 1.6	<0.01
Nm *ptsH::ptsH*	27.8 ± 1.3	0.34
Nm ptsH*	33.3 ± 1.4	<0.01
Nm *ccpA*	30.0 ± 1.3	0.19
Nm *ccpA_ptsH*	34.3 ± 1.0	<0.01

^1^ Data are presented as mean ± SD (*n* = 6). ^2^ *p* values were determined by one-way ANOVA and Holm-Sidak’s multiple comparison test.

## Data Availability

The datasets generated and analyzed during the current study are available from the corresponding author on reasonable request.

## Supplementary Materials

The following are available online at https://www.mdpi.com/2076-2607/9/3/466/s1, Table S1: Primers used in this study.

## References

[1] Deutscher J. (2008). The mechanisms of carbon catabolite repression in bacteria. Curr. Opin. Microbiol..

[2] Görke B., Stülke J. (2008). Carbon catabolite repression in bacteria: Many ways to make the most out of nutrients. Nat. Rev. Microbiol..

[3] Richardson A.R. (2019). Virulence and Metabolism. Microbiol. Spectr..

[4] Henkin T.M., Grundy F.J., Nicholson W.L., Chambliss G.H. (1991). Catabolite repression of α amylase gene expression in *Bacillus subtilis* involves a trans-acting gene product homologous to the *Escherichia coli lacl* and *galR* repressors. Mol. Microbiol..

[5] Weickert M.J., Chambliss G.H. (1990). Site-directed mutagenesis of a catabolite repression operator sequence in *Bacillus subtilis*. Proc. Natl. Acad. Sci. USA.

[6] Li C., Sun F., Cho H., Yelavarthi V., Sohn C., He C., Schneewind O., Bae T. (2010). CcpA mediates proline auxotrophy and is required for *Staphylococcus aureus* pathogenesis. J. Bacteriol..

[7] Deutscher J., Saier M.H. (1983). ATP-dependent protein kinase-catalyzed phosphorylation of a seryl residue in HPr, a phosphate carrier protein of the phosphotransferase system in *Streptococcus pyogenes*. Proc. Natl. Acad. Sci. USA.

[8] Schumacher M.A., Allen G.S., Diel M., Seidel G., Hillen W., Brennan R.G. (2004). Structural Basis for Allosteric Control of the Transcription Regulator CcpA by the Phosphoprotein HPr-Ser46-P. Cell.

[9] Leiba J., Hartmann T., Cluzel M.E., Cohen-Gonsaud M., Delolme F., Bischoff M., Molle V. (2012). A novel mode of regulation of the *Staphylococcus aureus* catabolite control protein A (CcpA) mediated by Stk1 protein phosphorylation. J. Biol. Chem..

[10] Ramstrom H., Sanglier S., Leize-Wagner E., Philippe C., Van Dorsselaer A., Haiech J. (2003). Properties and regulation of the bifunctional enzyme HPr kinase/phosphatase in *Bacillus subtilis*. J. Biol. Chem..

[11] Hengstenberg W., Penberthy W.K., Hill K.L., Morse M.L. (1969). Phosphotransferase system of *Staphylococcus aureus*: Its requirement for the accumulation and metabolism of galactosides. J. Bacteriol..

[12] Postma P.W., Lengeler J.W., Jacobson G.R. (1993). Phosphoenolpyruvate:carbohydrate phosphotransferase systems of bacteria. Microbiol. Rev..

[13] Deutscher J., Küster E., Bergstedt U., Charrier V., Hillen W. (1995). Protein kinase-dependent HPr/CcpA interaction links glycolytic activity to carbon catabolite repression in Gram-positive bacteria. Mol. Microbiol..

[14] Deutscher J., Francke C., Postma P.W. (2006). How Phosphotransferase System-Related Protein Phosphorylation Regulates Carbohydrate Metabolism in Bacteria. Microbiol. Mol. Biol. Rev..

[15] Seidl K., Stucki M., Ruegg M., Goerke C., Wolz C., Harris L., Berger-Bächi B., Bischoff M. (2006). *Staphylococcus aureus* CcpA affects virulence determinant production and antibiotic resistance. Antimicrob. Agents Chemother..

[16] Seidl K., Müller S., François P., Kriebitzsch C., Schrenzel J., Engelmann S., Bischoff M., Berger-Bächi B. (2009). Effect of a glucose impulse on the CcpA regulon in *Staphylococcus aureus*. BMC Microbiol..

[17] Reed J.M., Olson S., Brees D.F., Griffin C.E., Grove R.A., Davis P.J., Kachman S.D., Adamec J., Somerville G.A. (2018). Coordinated regulation of transcription by CcpA and the *Staphylococcus aureus* two-component system HptRS. PLoS ONE.

[18] Seidl K., Bischoff M., Berger-Bächi B. (2008). CcpA mediates the catabolite repression of *tst* in *Staphylococcus aureus*. Infect. Immun..

[19] Seidl K., Goerke C., Wolz C., Mack D., Berger-Bächi B., Bischoff M. (2008). *Staphylococcus aureus* CcpA affects biofilm formation. Infect. Immun..

[20] Halsey C.R., Lei S., Wax J.K., Lehman M.K., Nuxoll A.S., Steinke L., Sadykov M., Powers R., Fey P.D. (2017). Amino Acid Catabolism in *Staphylococcus aureus* and the Function of Carbon Catabolite Repression. mBio.

[21] Nuxoll A.S., Halouska S.M., Sadykov M.R., Hanke M.L., Bayles K.W., Kielian T., Powers R., Fey P.D. (2012). CcpA regulates arginine biosynthesis in *Staphylococcus aureus* through repression of proline catabolism. PLoS Pathog..

[22] Cramton S.E., Gerke C., Schnell N.F., Nichols W.W., Götz F. (1999). The intercellular adhesion (*ica*) locus is present in *Staphylococcus aureus* and is required for biofilm formation. Infect. Immun..

[23] Mann E.E., Rice K.C., Boles B.R., Endres J.L., Ranjit D., Chandramohan L., Tsang L.H., Smeltzer M.S., Horswill A.R., Bayles K.W. (2009). Modulation of eDNA release and degradation affects *Staphylococcus aureus* biofilm maturation. PLoS ONE.

[24] Bischoff M., Wonnenberg B., Nippe N., Nyffenegger-Jann N.J., Voss M., Beisswenger C., Sunderkötter C., Molle V., Dinh Q.T., Lammert F. (2017). CcpA Affects Infectivity of *Staphylococcus aureus* in a Hyperglycemic Environment. Front. Cell. Infect. Microbiol..

[25] Liao X., Yang F., Wang R., He X., Li H., Kao R.Y., Xia W., Sun H. (2017). Identification of Catabolite Control Protein A from *Staphylococcus aureus* as a Target of Silver Ions. Chem. Sci..

[26] Hartmann T., Zhang B., Baronian G., Schulthess B., Homerova D., Grubmüller S., Kutzner E., Gaupp R., Bertram R., Powers R. (2013). Catabolite control protein E (CcpE) is a LysR-type transcriptional regulator of tricarboxylic acid cycle activity in *Staphylococcus aureus*. J. Biol. Chem..

[27] Giachino P., Engelmann S., Bischoff M. (2001). Sigma(B) activity depends on RsbU in *Staphylococcus aureus*. J. Bacteriol..

[28] Monk I.R., Shah I.M., Xu M., Tan M.W., Foster T.J. (2012). Transforming the untransformable: Application of direct transformation to manipulate genetically *Staphylococcus aureus* and *Staphylococcus epidermidis*. mBio.

[29] Leibig M., Krismer B., Kolb M., Friede A., Gotz F., Bertram R. (2008). Marker removal in staphylococci via Cre recombinase and different lox sites. Appl. Environ. Microbiol..

[30] Duthie E.S. (1952). Variation in the antigenic composition of staphylococcal coagulase. J. Gen. Microbiol..

[31] Kreiswirth B.N., Lofdahl S., Betley M.J., O’Reilly M., Schlievert P.M., Bergdoll M.S., Novick R.P. (1983). The toxic shock syndrome exotoxin structural gene is not detectably transmitted by a prophage. Nature.

[32] Iordanescu S., Surdeanu M. (1976). Two Restriction and Modification Systems in *Staphylococcus aureus* NCTC8325. Microbiology.

[33] Gaupp R., Wirf J., Wonnenberg B., Biegel T., Eisenbeis J., Graham J., Herrmann M., Lee C.Y., Beisswenger C., Wolz C. (2016). RpiRc Is a Pleiotropic Effector of Virulence Determinant Synthesis and Attenuates Pathogenicity in *Staphylococcus aureus*. Infect. Immun..

[34] Beenken K.E., Blevins J.S., Smeltzer M.S. (2003). Mutation of *sarA* in *Staphylococcus aureus* limits biofilm formation. Infect. Immun..

[35] Zhu Y., Nandakumar R., Sadykov M.R., Madayiputhiya N., Luong T.T., Gaupp R., Lee C.Y., Somerville G.A. (2011). RpiR homologues may link S*taphylococcus aureus* RNAIII synthesis and pentose phosphate pathway regulation. J. Bacteriol..

[36] Rupp M.E., Ulphani J.S., Fey P.D., Bartscht K., Mack D. (1999). Characterization of the importance of polysaccharide intercellular adhesin/hemagglutinin of *Staphylococcus epidermidis* in the pathogenesis of biomaterial-based infection in a mouse foreign body infection model. Infect. Immun..

[37] Ferreira M.T., Manso A.S., Gaspar P., Pinho M.G., Neves A.R. (2013). Effect of oxygen on glucose metabolism: Utilization of lactate in *Staphylococcus aureus* as revealed by in vivo NMR studies. PLoS ONE.

[38] Vitko N.P., Grosser M.R., Khatri D., Lance T.R., Richardson A.R. (2016). Expanded glucose import capability affords *Staphylococcus aureus* optimized glycolytic flux during infection. mBio.

[39] Gill S.R., Fouts D.E., Archer G.L., Mongodin E.F., Deboy R.T., Ravel J., Paulsen I.T., Kolonay J.F., Brinkac L., Beanan M. (2005). Insights on evolution of virulence and resistance from the complete genome analysis of an early methicillin-resistant *Staphylococcus aureus* strain and a biofilm-producing methicillin-resistant *Staphylococcus epidermidis* strain. J. Bacteriol..

[40] Deutscher J., Herro R., Bourand A., Mijakovic I., Poncet S. (2005). P-Ser-HPr--a link between carbon metabolism and the virulence of some pathogenic bacteria. Biochim. Biophys. Acta.

[41] Pullen K., Rajagopal P., Branchini B.R., Huffine M.E., Reizer J., Saier M.H., Scholtz J.M., Klevit R.E. (1995). Phosphorylation of serine-46 in HPr, a key regulatory protein in bacteria, results in stabilization of its solution structure. Protein Sci. A Publ. Protein Soc..

[42] Thapar R., Nicholson E.M., Rajagopal P., Waygood E.B., Scholtz J.M., Klevit R.E. (1996). Influence of N-cap mutations on the structure and stability of *Escherichia coli* HPr. Biochemistry.

[43] Sadykov M.R., Hartmann T., Mattes T.A., Hiatt M., Jann N.J., Zhu Y., Ledala N., Landmann R., Herrmann M., Rohde H. (2011). CcpA coordinates central metabolism and biofilm formation *in Staphylococcus epidermidis*. Microbiology.

[44] Hartmann T., Baronian G., Nippe N., Voss M., Schulthess B., Wolz C., Eisenbeis J., Schmidt-Hohagen K., Gaupp R., Sunderkötter C. (2014). The catabolite control protein E (CcpE) affects virulence determinant production and pathogenesis of *Staphylococcus aureus*. J. Biol. Chem..

[45] Ding Y., Liu X., Chen F., Di H., Xu B., Zhou L., Deng X., Wu M., Yang C.G., Lan L. (2014). Metabolic sensor governing bacterial virulence in *Staphylococcus aureus*. Proc. Natl. Acad. Sci. USA.

[46] Balasubramanian D., Ohneck E.A., Chapman J., Weiss A., Kim M.K., Reyes-Robles T., Zhong J., Shaw L.N., Lun D.S., Ueberheide B. (2016). *Staphylococcus aureus* coordinates leukocidin expression and pathogenesis by sensing metabolic fluxes via RpiRc. mBio.

[47] Montgomery C.P., Boyle-Vavra S., Roux A., Ebine K., Sonenshein A.L., Daum R.S. (2012). CodY deletion enhances in vivo virulence of community-associated methicillin-resistant *Staphylococcus aureus* clone USA300. Infect. Immun..

[48] Ha J.H., Hauk P., Cho K., Eo Y., Ma X., Stephens K., Cha S., Jeong M., Suh J.Y., Sintim H.O. (2018). Evidence of link between quorum sensing and sugar metabolism in *Escherichia coli* revealed via cocrystal structures of LsrK and HPr. Sci. Adv..

