# Supplementary material for: Impact of the Histidine-Containing Phosphocarrier Protein HPr on Carbon Metabolism and Virulence in Staphylococcus aureus
Source: Microorganisms. 2021 Feb 24;9(3):466. doi: 10.3390/microorganisms9030466 (PMC7996215; doi:10.3390/microorganisms9030466)
Supplement: Supplementary file 1 [file microorganisms-09-00466-s001.pdf]

Supplementary Material

Impact of the histidine-containing phosphocarrier protein HPr on carbon metabolism and virulence in *Staphylococcus aureus*

Linda Pätzold [1], Anne-Christine Brausch [1], Evelyn Kirch [1], Lisa Zimmer [1], Greg A. Somerville [2], Markus Bischoff [1,*], and Rosmarie Gaupp [1]

[1] Institute of Medical Microbiology and Hygiene, Saarland University, Homburg/Saar, Germany
[2] School of Veterinary Medicine and Biomedical Sciences, University of Nebraska, Lincoln, Nebraska, USA
* Correspondence: markus.bischoff@uks.eu; Tel.: +49 6841 162 39 63

Supplementary Table 1: Primers used in this study.

Primer	Direction	Sequence (5′-3′) [1]
**Cloning primer**		
MBH20	Rev	gtcggtacCTCCACCTATATCTAAAGTACG
MBH86	For.	GGTGTTATGAGCCTTGGTG
MBH94	Rev.	ctcgaaTTCCATAATTACATTTCTCCTTCG
MBH112	For.	gtcggtACCTTGAAGATGGTTTGATGAC
MBH113	For.	gtcggatCCATTCAAGCAATCAGTGATGTC
MBH114	Rev.	ggatctAGAATGGATTCATTTCTTCAGGC
MBH427	For.	gtcgaattCTGAGTTTTTATATATGGGTCGTG
MBH428	Rev.	gtcggtacCTACATTTTGTTAATGACACATAGTTG
MBH484	Rev.	CCAACACCAAGGCTCATAACACCCATGATTGcTTTTAAGTTTACTTTCTTACC
MBH485	For.	GAATTATGTCTGCAGCAGCAATTCAAGGTAGTC
**qRT-PCR primer**		
citB	For.	CAAGATCATCAAGTGCCTATTCGT
citB	Rev.	CGTGATTACCACGTCTTGAACC
gyrB	For.	GACTGATGCCGATGTGGA
gyrB	Rev.	AACGGTGGCTGTGCAATA
hla	For.	AACCCGGTATATGGCAATCAACT
hla	Rev.	CTGCTGCTTTCATAGAGCCATTT
icaA	For.	CTGGCGCAGTCAATACTATTTCGGGTGTCT
icaA	Rev.	GACCTCCCAATGTTTCTGGAACCAACATCC
pckA	For.	CACGGCTGGAATAAAAACGG
pckA	Rev.	TGCATAGCAGCCACCTTCG

[1] Small letters represent nucleotides that do not fit with the target sequence. Restriction sites used for cloning are underlined.
